# Assistance Dogs for People with Younger (Early)-Onset Dementia: The Family Carer’s Experience

**DOI:** 10.3390/ani13050777

**Published:** 2023-02-21

**Authors:** Genée Marks, Keith R. McVilly

**Affiliations:** 1 School of Education, Deakin University, Geelong, VIC 3220, Australia; 2 School of Social & Political Sciences, University of Melbourne, Parkville, VIC 3010, Australia

**Keywords:** Alzheimer’s assistance dogs, animal-assisted therapy, human–animal bond, younger onset dementia, early onset dementia, carers, wellbeing

## Abstract

**Simple Summary:**

There is growing evidence for the efficacy of trained assistance dogs to enhance the health and wellbeing of people in a variety of circumstances, including those with dementia. However, little is understood about the effect of assistance dogs on the wellbeing of family members providing care to those with dementia. Our study included interviews conducted with family carers over a period of up to two years. We found that in addition to the positive effects on those with younger-onset dementia (YOD), family carers also reported many benefits to the wider family and their own wellbeing. In many instances, these benefits persisted after the family member with dementia needed to go into out-of-home care. However, to establish and sustain these arrangements and ensure the wellbeing of both family members and the dogs, attention needs to be given to the family’s living arrangements and the resources (including financial) available to families.

**Abstract:**

There is growing evidence for trained assistance dogs promoting the health, wellbeing, and quality of life of people in a variety of circumstances, including for those with dementia. Little is known about people with younger (early)-onset dementia (YOD) and family carers. As part of a larger study involving 14 people with YOD matched with trained assistance dogs over a two-year period, we report analyses of interviews with 10 family carers conducted on multiple occasions investigating their experience with an assistance dog. Interviews were recorded, transcribed and subjected to inductive thematic analysis. They told a range of experiences; the good and the challenging. Findings fell into three areas: the human–animal bond; relationship dynamics; and responsibility for caring. Concerns were raised with respect to the resources required of carers together with the financial resources needed to support an assistance dog. The study concludes that trained assistance dogs can play an important role promoting the health and wellbeing of both people with YOD and of their family carers. However, support needs to be in place as the circumstances of the family member with YOD changes and the role of the assistance dog as part of the family also changes. Practical (financial) support of a scheme such as the Australian National Disability Insurance Scheme (NDIS) could be important to sustaining such support.

## 1. Introduction

In Australia, there are an estimated 487,500 people living with dementia [1], which is the second leading cause of death in Australia [2]. Notably, rates of dementia are on the rise in Australia, with an average of 250 being reported every day, and an expected 318 per day by 2025. Similarly, in Japan, USA and UK it is expected that 1 in 3 people will develop dementia in their lifetime with the most common being Alzheimer’s [3,4]. Furthermore, research commissioned by Dementia Australia estimate there to be about 1.6 million Australians involved in the care of someone living with dementia. In the USA, this figure is estimated to be over 11 million [3].

While the vast majority of people with dementia are over 65 years old when diagnosed, 5% to 6% of people with dementia will have younger-onset dementia (YOD), also known as early-onset dementia (EOD) [5]. YOD is used to describe any form of dementia diagnosed in people who are younger than 65 years old. It is far less common than dementia diagnosed when an individual is over 65 and has been diagnosed in people as young as in their 30s. 

When diagnosed, these individuals are typically living at home with a partner and often have parental responsibilities for their children or younger family. Busted, Nielsen, & Birkelund [6] report that individuals with YOD experience a range of emotions such as fear of a loss of control over oneself, becoming a burden on their family, fear of a humiliating future, and a loss of their sense of self and who they are as a partner, parent and person. Bannon et al. [7] report that those diagnosed with YOD and their family carers experience a range of stressors that impact their health and wellbeing. These include: difficulty understanding the diagnosis and/or disease trajectory; life being “turned upside down”; a sense of loss of anticipated future and “golden years” together; negative emotions such as anxiety, anger, grief, despair, and hopelessness; feeling overwhelmed with information as they connect with support services; and a lack of acceptance or denial of one partner interfering in a couple’s ability to adjust together. These can be exacerbated as the person with YOD deteriorates cognitively and physically (including loss of the ability to speak, walk and loss of continence), and their behaviors become erratic and at times aggressive.

As there is no known cure for dementia, the primary focus of intervention and support is to maintain the person’s quality of life. This is achieved through a combination of activities designed to maximize their engagement with and independence in daily activities, sustain recognition of their immediate environment and significant others, promote social engagement, and stabilize mood and behavior. Importantly, intervention and support are also needed to foster a safe living environment. Johannessen, Helvik, Engedal, and Thorsen [8] report that as part of a holistic intervention, it is also very important to address the psychological and practical needs of partners providing care through individualized interdisciplinary support throughout the progression of YOD. The need to focus on the support of partners providing care is further highlighted by Werner, Spiegelman and Turgeman [9] who identify several types of stigma adversely impacting carers of those with YOD; including public stigma reflected in social distancing and discrimination by others in the community, courtesy stigma where relatives and friends distance themselves, structural stigma where professionals decline to provide services, and personal negative emotions where partners report feeling embarrassment and shame and as a consequence guilt. 

Trained assistance dogs might offer one means of effective non-pharmacological intervention for those living with dementia and potentially also for family members providing care [10,11,12]. Notably, potential benefits have been observed for both the person with dementia and their family, particularly in relation to the human–animal bond (i.e., the positive roles that animals can play in the integrated health of individuals, families and communities), relationship dynamics and the responsibilities of caring [13]. Such findings reflect research in the general population, where interactions and activities with dogs have been shown to both promote behaviors known to increase wellbeing (e.g., walking), and to more directly affect human wellbeing [14,15,16]. In particular, tactile interactions and playing with a dog have been found to be beneficial for hedonic wellbeing (i.e., that which is achieved through pleasurable experiences), while participating in activities such as dog training and caring for a dog have been found to contribute to eudemonic wellbeing (i.e., that which is achieved through pursuing self-improvement and realizing ones’ best self) [17]. 

This study was informed by a systematic review of peer-reviewed literature [10]. The review focused on the efficacy of trained assistance dogs as an intervention and support for people experiencing dementia. However, the review was unable to identify literature specifically addressing the health and wellbeing of those with YOD, nor was it able to identify literature documenting the experience of family carers supporting a person with YOD. Since the publication of Marks and McVilly [10], Santaniello et al. [11] have conducted a 7-year retrospective analysis of 127 Alzheimer’s patients and concluded that animal-assisted therapy involving dogs has promising potential in its efficacy in the treatment of cognitive deficits arising from Alzheimer’s disease. Furthermore, Ritchie et al. [13] have undertaken an initial investigation based on the reports of carers exploring the effectiveness of canine-assisted interventions for people with dementia in Scotland. However, there is still no evident literature focusing on the experience and effect of trained dementia assistance dogs specifically for people with YOD and their family carers. 

Consequently, the project of which this study forms a part was among the first properly constituted longitudinal research into the efficacy of assistance dogs for people with YOD. It provided a framework within which to develop training for assistance dogs for use with people with YOD, safely place the dogs and investigate their impact on the lives of both people with YOD and on their families. This study, the first from a larger project, reports on the experience and impact of trained assistance dogs on family members in the carer role for persons with YOD.

## 2. Methodology

### 2.1. The Context for the Study

The larger project was designed to evaluate the effects of assistance dogs on the quality of life, health and wellbeing of people with YOD and family members providing support and care. The methodology was developed which documented the extent to which people with YOD, if provided with a trained dementia assistance dog, would be able to sustain their physical and emotional wellbeing, and self-manage the behavioral sequelae associated with dementia. Furthermore, the project monitored the engagement of participants with YOD in a range of practical everyday tasks that fostered physical health, together with their exercise of choice and self-determination. 

Finally, the project examined the impact of the assistance dog program on the homeostatic wellbeing of a family member, identified as the ‘primary carer’ for the person with YOD. The term homeostatic wellbeing is derived from an analogy between the physiological management of, for example, body temperature by the autonomic nervous system and the maintenance of subjective wellbeing theorized to be managed as an interplay of neurological systems the result of which is experienced in mood states such as happiness and sadness [18].

Both quantitative and qualitative data were gathered, though in this paper we focus on the qualitative analysis of the family members’ narratives collected by means of individual semi-structured interview on multiple occasions. Data collection took place over a period of up to two years, depending upon the progression of the disease for individual participants and their family circumstances. As the YOD progressed, some participants declined in their ability to contribute to the interview process.

### 2.2. Ethics

The protocol for this research was reviewed and approved by the supervising institution’s Human Research Ethics Committee (HREC); approval number 1647054. An application was also made to the host institution’s Animal Research Ethics Committee but was subsequently exempted from the need for formal approval with the Animal Ethics Committee satisfied that the wellbeing of the dogs was sufficiently addressed in the human research ethics protocol; including pre-checks on the prospective families with which the dogs were to be placed and the physical environment of the house and garden, follow up wellbeing checks were arranged for post placement, and a protocol for withdrawal of the dogs agreed to with families prior to placement.

All data reported in the final analysis, such as descriptions and interpretations of induced themes, have been anonymized to protect the privacy of participants and carers. However, it is acknowledged that since several the participants have chosen to participate in documentaries, television broadcasts, newspaper reports, and present at conferences, as well as five families living in regional or rural communities, it may be possible for some participants to be identified. These participants and their cares were aware of this risk at the time data were collected and gave consent accordingly.

### 2.3. Participants

The carers who are the primary focus of the current study were the partners and adult children of participants with YOD. Participants with YOD and their carer/partners were recruited into the program and matched with assistance dogs with the support of a national dementia advocacy organization. The protocol for this recruitment and matching process was developed by the assistance dogs training organization, with reference to the protocols used to match persons who are blind or with low vision to Seeing-eye (Guide) dogs. Importantly, this protocol also took into account the safety and wellbeing of the dog. 

Participants with dementia were medically diagnosed with YOD, occurring prior to 65 years of age, and of the Alzheimer’s type. There were five women and five men. The mean age of participants with dementia was 61 years 1 month (ranging from 53 years to 65 years of age), and seven of the participants were urban residents, and the remainder lived in a regional or rural area. Their carers were predominantly life partners (*n* = 12), with the occasional adult offspring also providing primary carer support (*n* = 2).

Three of these triads (i.e., the person with YOD, the carer and the assistance dog) subsequently left the program: one to travel overseas for a year, one because of later discovering dog hair allergies, and a third prior to the allocation of their dog. A fourth triad left the program when the dog needed to be repatriated due to ill health. Of the 10 remaining triads, whose data form the basis of the current analyses, three had a partner with dementia who went into care during the period of the research, and the final seven remained at home for the full two-year duration of the project.

While the primary focus of the current study is on the carers, the experiences and consequently the stories of the carers, their partners with dementia, and the dogs were intertwined. The experience of the participants with dementia often gave important context to that of the carers’ reported experiences. This intertwining of experience is consequently reflected in the data and subsequent analysis. As noted earlier, pseudonyms have been used to maintain participant anonymity. In the brief descriptions that follow, the pseudonym of the person with YOD comes first, followed by that of the family carer and then the assistance dog. 

#### 2.3.1. Participants who Went into Care during the Program

Case study #1—Sandra, Peter and their dog Aspen. 

Sandra and her partner and carer Peter lived in the outer suburbs of a large city and withdrew from the program between the second and third interview because Sandra went into care and could not take the dog. The family spent considerable time equivocating about this, but Peter needed to seek full-time employment, and did not feel he could commit to providing support for the dog, consequently Aspen was returned to the program. 

Case study #2—Cathy, Michael and their dog Beth. 

The domestic situation for this couple was very different to that of other participants. The family lived in an apartment that required access by a keyed password to the building, and then to the lift. This made it impossible for Cathy to take the dog out by herself, although whether or not she would have been able to do so in different living circumstances was unclear. Cathy went into care, but the family kept Beth and Michael took Beth to see Cathy at the nursing home regularly. 

Case study #3—Stephen, Michelle and their dog Andrew.

Stephen and Michelle lived in an inner suburb of a large city. At the time of the first interview, Stephen’s dementia was already quite advanced, and Michelle was struggling to provide the necessary care. It was causing her a great deal of anxiety. It was reported that Andrew was too much stress and worry for Michelle to handle as Stephen’s support needs increased and consequently Andrew was returned to the program.

#### 2.3.2. Participants who Were in the Program for the Duration of the Interview Period (Two Years)

Case study #4—Jason, Annabelle and their dog Maxwell. 

Jason and his partner Annabelle, were both in their 60s and had been together for over 30 years, were both tertiary educated, and had both worked in professional roles. They lived in a regional center and were very active socially and politically. They had additional paid care-workers to help meet Jason’s relatively high support needs, and they made regular use of respite care for several weeks at a time.

Case study #5—James, Sue and their dog George. 

James and his partner Sue had been married for forty years. James completed form 3 at high school and worked in retail until being diagnosed with YOD. This couple lived in a regional center, and had adult children and grandchildren, living both locally and in other states. Sue still worked almost full-time, and James was home alone most of the day. They also minded grandchildren on a regular basis, but not when James was home alone. 

Case study #6—Linda, Jeff and their dog Ellie. 

Jeff and Linda lived in a large regional city. They had three adult daughters who did not live at home. Both were very physically active. The couple had lived and worked overseas, and Jeff was of a European migrant family. This couple had always had dogs in their households.

Case study #7—Marcus, Lynette and their dog Jasper.

Marcus and his partner Lynette lived on the outskirts of a small rural town located on an island, where they had moved recently following Marcus’ diagnosis. They both had teaching backgrounds in different disciplines, and had adult sons, and several grandchildren. They were both in their late 50s at the time of the first interview and had been married for nearly 40 years. Marcus came from a family that had never had dogs, so having a dog of his own was a new experience for him. 

Case study #8—Cassie, Chris and their dog Raymond.

Cassie and Chris lived in a rural town, where they had lived for many years. Chris had been in a leadership role in a large regional school, was a highly qualified teacher with several master’s level qualifications, and at the time of the first two interviews, was still working part-time within the school, despite his memory loss, and was still driving a car. Cassie worked in a government department at a senior level, but by the third interview had taken some extended leave. Close family ties existed, with Chris living in a nearby regional town, with one adult son living locally and working nearby. 

Case study #9—Hugh, Marlene and their dog Jones.

Hugh and his wife Marlene emigrated to Australia some decades earlier. They lived in a unit on the outskirts of Melbourne, and their adult son lived nearby. Hugh had been a tradesman and an athlete when he was younger. Hugh’s dementia was very advanced before the dog was allocated. At the start of their involvement in the research, Hugh’s speech was very difficult to understand. He had trouble moving around the room and maintaining balance, and his sight was bad. 

Case study #10—Sandy, Damian and their dog Jessie.

Sandy was in her 60s, and Damian in his early 70s. They had two adult children and had been married for over 40 years. At the time of the first interview, they were living in their long-standing family home in the outer suburbs of a large city but were in the process of transitioning to a property on the outskirts of a small rural town. Sandy was already spending several days a week at the property, while Damian was attending to business in the city. By the time of the third interview, they were both permanently living at the rural property. 

#### 2.3.3. The Assistance Dogs

Assistance dogs have been defined in various jurisdictions across Europe, the USA and Australia [19,20]. In the USA, they are referred to as ‘service animals’ in the Americans with Disability Act [21]. They are distinguished from ‘therapy dogs’ in that assistance dogs are matched and placed with specific people for prolonged periods to help with identified needs. This is in contrast to therapy dogs that augment the work of practitioners across multiple clients for shorter-term interventions. They are also to be distinguished from ‘companion dogs’. In Australia, assistance animals, including dogs, are defined in the Commonwealth Disability Discrimination Act [22], and there are state-based registration schemes. Similarly, in New Zealand, discrimination against persons relying on or training an Assistance Dog may be considered a breach of the Human Rights Act 1993 [23] (S21) and/or the Dog Control Act 1996 [24].

Generally, before being registered, an assistance dog must first pass a ‘public access test’ (i.e., the minimum standard an assistance animal must meet to be considered safe and effective in accessing public places and public passenger vehicles). Notably, as trained and registered assistance dogs, the dogs in the current project were able to accompany their owners on public transport and into cafés, restaurants, cinemas, and other public places where other dogs (pets) would not ordinarily be allowed. 

The dogs were sourced as puppies from breeding stock originally intended to be trained as Seeing-eye (guide) dogs for the blind and people of low vision. They were then trained by an organization experienced in the training of Seeing-eye (guide) dogs for people who are blind or of low vision. The training process took place when the dogs were around 12–14 months old. At that stage all of the dogs had passed their first year of basic Seeing-eye (guide) dog training, obedience and general management. Both the training and the placement protocols took into account what has subsequently been referred to by Salmon et al. [25] as “the animal’s right to flourish or to lead a life worth living where mutual benefit exists for both animal and human” (p. 3250). 

The protocol for training was developed in consultation with a national organization involved in the support of people with dementia, including those with YOD. Additionally, the dog trainers underwent dementia-specific training and consulted with a project reference group comprising people with YOD. Training tasks included: head on lap (on demand); fetch the carer; dog follows participant throughout the house unless commanded otherwise; find object (mobile telephone, glasses, etc.); stop at kerb; and tug and response games (i.e., where the dog waits for a command to take a toy and then waits for a commend to release the toy). All dogs were trained to walk in harness, and in the usual skills expected of Seeing-eye (guide) dogs for the blind.

### 2.4. Data Collection and Analysis

This project utilized a multiple single case study approach. For the current study, all data were collected by semi-structured interviews conducted in the participants’ homes. All interviews were conducted by the first author. While participants were typically interviewed in the presence of their family carer because of their communication support needs, family carers were usually interviewed separately. The current analyses focus on the qualitative data arising from the interviews with family carers.

Interviews were conducted at three points: time 1 baseline assessment, which occurred about 6 weeks into the program once suitability had been ascertained and before participant-specific dog placement and training began (at between weeks 7 and 10); time 2 at about week 24, once dogs had been placed, participants trained, and participants, carers and dogs had been assessed as suitable and ‘graduated’; and time 3, at least one year later, which in some cases took place at up to 18 months after the original placement. Interviews were audio recorded and transcribed for analysis.

Interview transcripts were first analyzed using Inductive Thematic Analysis to pinpoint recurrent themes within and across the case studies. The coding of emergent themes within the thematic analysis enabled an understanding of their frequency and generated a complex picture of the value of dementia assistance dogs and associated challenges. The extent of these analyses took into account the relatively small sample size. However, because the data from the various case studies were gathered over a period of up to two years, it was possible to develop rich insight into the participants’ circumstances. Data were initially analyzed by the first author and the formulation then reviewed by the second author. Any discrepancies were conciliated by means of discussion and the coding reviewed as required.

The results were subsequently organized deductively, informed by the findings of Ritchie et al. [13], who observed that outcomes (for both participants and their carers) fell into three key areas: the human–animal bond; relationship dynamics; and responsibility for caring. We also found these categories emerged in our analysis. Additionally, we found evidence for the positive effects the assistance dogs had on carer wellbeing, which extended beyond the time when the person with dementia needed to move into out-of-home care. 

## 3. Results and Discussion

### 3.1. Human–Animal Bond

The bond between human and animal is regarded as both mutualistic and dynamic in that it influences the behavior and wellbeing of both the animal and the human. [26]. Over history, the nature of this bond has evolved, especially in relation to the bond between the dog and humans. As Ritchie et al. [13] discovered, we also found that each individual involved with the project, whether they are a partner/carer, family member, or people with dementia, developed their own, individually diverse relationship and bond with the assistance dog. These bonds were highly variable, not only between the carer/family members, and person with dementia, but also across the project within different families. 

An example of the way the human–animal bond worked was seen when Stephen (CS#3) went into full-time care, but his partner Michelle continued to bring Andrew (the assistance dog) to visit Stephen in the care facility. Stephen, it transpired, believed he was an employee of the care facility, and that he was earning money to support the dog and his family. Michelle reported “*It makes him feel important!*” This gave Stephen a renewed sense of purpose through his bond with the dog. Indeed, shortly before the time of the interview, Stephen had been having great difficulties with being in care, and according to his support worker, having the dog visit had been seen as one of the calming influences. As Michelle noted: “*The bond between them. You can see it’s great*”. The role of the dog in providing companionship, and the opportunity for tactile contact, were seen as highly significant in supporting Stephen. More pertinently, for this paper, seeing Stephen develop a human–animal bond with his assistance dog, and accordingly, a sense of purpose, gave his partner Michelle a greater bond with the dog, as she considered both she and the dog were working in consort, drawing on their human–animal bond, to strengthen Stephen’s sense of wellbeing.

The human–animal bond emerged as significant for this family in other ways too. The role of the dog with the remaining family is worthy of comment, as the dog seemed to be playing a part in assisting the family to grieve, and to move into a different phase in their lives. Michelle observed, that for the family (including their adult children in their mid-twenties still living at home): “*I think [the dog] is replacing Stephen. He’s helping the three of us at home, the children coping, and me coping. We’ve all become very attached to him*”. Michelle further reflected:

*So, it helps them too. Definitely. So, he virtually has not replaced Stephen, but they can, instead of being upset, they can cuddle [the dog] and interact. He has a special role for each one of us. He knows I’m, like, the mum, Stephen’s the dad that I cuddle, L is his sister, so they’ll cuddle a lot and L kisses them a lot, so they’re snuggle bunnies, and D I’m allowed to play rough and play games with D. So, he has a role for each of us.*

Responses like this resonate with the discoveries of Ritchie et al. [13], who note that not only did the dogs reduce the stress of the carer’s role, but also because (in the case of Michelle as a carer), the bond between the carer and the dog was “*based on trust and appreciation of shared responsibility in the caring role*”.

### 3.2. Relationship Dynamic

Relationship dynamics (i.e., the patterns of behavior between people and how they relate, interact and communicate with each other) varied, as already noted, across the 10 triads. These relationships often changed when the participant with YOD went into care, and this was commented on across all but one of cases of participants going into care full time. Before Cathy (CS#2) went into care, for example, her assistance dog Beth was largely treated as a pet in the home and was seldom in a harness. Once Cathy went into care, however, the dog was taken into the care facility nearly every day. Although Michael (Cathy’s carer) noted that Cathy had deteriorated very rapidly, she still regularly asked for the dog, and Michael ensured that the dog and participant had the tactile contact that they both enjoyed. Michael described that “Beth [the dog] *nuzzles up to her. Cathy tries to pet her, but she has no coordination. I take her hand and help her pet the dog. Beth sits at her feet*”. He also reported that other people in the care facility loved seeing the dog, and although she is not the only dog that visits: “*they all love to see her, and she talks to them. Beth is delighted*”.

For Michael, as a carer, this enabled a relationship dynamic between him and Cathy that had been lost when she was struggling at home with her dementia. Importantly too, for this family, the assistance dog provided family support in the transition process. Michael commented:

“*I have a 29-year-old daughter [who lives at home]. I think she has struggled seeing her mother decline. But she has a great rapport with the dog. Beth is a distraction when I look after her. The three of us sit in the courtyard in the sunshine. It’s become part of the relationship*”.

It was reported that the dog did her best work when Cathy went into care and the family was struggling emotionally to deal with the loss. This sort of shifting dynamic between dog and carers/family members was not uncommon across the project.

As the program progressed, three participants moved from home into care. Subsequently, the assistance dog played some unexpected roles in supporting the family in the transition process. This was not possible in the case of Peter (the carer) (CS#1) having to return to work full time, which meant there would have been nobody home with the dog most of the time and consequently Aspen needed to be returned to the program. In the other two cases where the participant went into care (CS2 & #3), as reported by the family members, the dog played a significant role for the family both in connecting them to the participant and adjusting to their changing circumstances.

Michelle (CS#3) for example, observed that with Stephen moving into full-time care, their relationship had changed for the better, following her difficulty in supporting his deteriorating condition, which included incontinence. She felt that at that point, she “*sort of fell in a heap*”. With the additional support of the staff at the care facility, and the opportunity for Michelle to take over the management and responsibility of the dog, Michelle observed: “*our relationship has completely changed again, like we’re happy to see each other*”. As noted earlier, having the dog still living with them had an important impact on this family, as the dog appeared to be aware of the family’s grief and provided each of the family members with individual support dealing with this, supporting the grieving process for the adult children in the family, as well as for Michelle. Ritchie et al. [13] also noted in their project that the dog provided “a vital source of support to the spouse” when the partner passed away, facilitating the process of dealing with grief.

### 3.3. Responsibility for Caring

The shared nature of the responsibility for caring between assistance dogs and family members providing support is particularly notable. Four family carers commented that the dogs maintaining a check on the participants with YOD provided reassurance to carers, and in three of these cases, they observed that they felt like they formed a care-giving team with the dog. Qualitative feedback from several of the carers confirmed they felt much greater confidence about the participants with dementia going out alone or being left alone in the house. This applied to both those participants who went into care during the program (specifically Sandra, Cathy, and Stephen), and those who stayed in the program, and in their homes, for the duration (Jason, James, Linda, Marcus, Cassie, Hugh, and Sandy). Others expressed this confidence in relation to leaving the home alone.

When Lynette (CS#7) was first interviewed, she was very depressed about the wellbeing of Marcus, and her ability to provide him with the necessary support. They had only recently moved to the region, and at that stage did not know people who would visit regularly. She expressed hope that Marcus, who was a very shy man, would go out visiting once he had the dog, to help deal with his loneliness when she was at work. She explained why they moved to the island: “*It was a place for Marcus to be happier in … A place for Marcus to be safe. You know, if he does start losing his way, which he doesn’t at all at the moment, it’s an island* …” Lynette was lucid and reflective, and expressed her own sadness and grief, as well as reflecting on that of Marcus. She concluded: “*And so I guess in terms of the dog, you know for me to know that he’s got that company at home, and it’s going to lessen his senses of grief and guilt and whatever.*” 

At the time of the second interview, Lynette observed: “*I think they’re doing very well together. They have a good bond*”. She also reiterated that Marcus and the dog spent considerable time having “*long beach adventure walks*” and noted that the three of them went to coffee shops and restaurants and went out visiting. When asked what was most important about Marcus having the dog, Lynette replied: “*Look I think it’s mostly companionship and confidence. Those two things*”. She added: “*Well, I like the fact that when I go out to work that Marcus feels much more, much less lonely. Much more like he’s got … Having the dog has given him … Sometimes gives him purpose, but also, it’s just than companionship*”. Knowing that Marcus had this companionship and support had meant that Lynette’s depression had decreased, because the bond between Marcus and the dog had relieved her of some of the responsibility of caring.

Similarly, Sue (CS#5), who was still working three days a week, mentioned that she felt much safer about James out walking by himself, and being home alone when she was at work, now that he had the dog. In particular, she drew attention to the concern about James wandering out in front of cars when he went to cross the road, and she emphasized: “*The biggest thing for me is that I know, I’m not worried when James is away from home*”, and she commented of George, the dog: “*He’s just such a blessing, such a blessing … It gets him out and moving, it gives me security and confidence. I’ve cut down my hours, but I still work 3 days a week, so yeah, when I’m at work*”.

In another case, Jeff (CS#6), for example, felt that prior to having the dog, he was uncertain about Linda going walking by herself to the supermarket, but she would now regularly go out shopping by herself, and return safely. By the end of the program, Jeff was so confident about Linda’s ability to manage alone with the dog, that he was going away on overnight trips, and planning a fortnight away leaving Linda by herself. Linda was confident that she would cope, and Jeff felt she would too. Jeff observed with some relief that now he felt that he was not alone in doing the caring, and that rather being the two of them trying to manage day-to-day, they were now “*a team of three*”.

### 3.4. Carer Wellbeing

Of particular interest in this research was the impact of the presence of the assistance dogs on the wellbeing of the carers. While it was originally envisaged that this would be measured quantitatively, no clear consistent patterns were apparent in the data, possibly due to the heterogeneity of the families and their circumstances and the relatively small sample size. This reinforced the importance of the qualitative data and the narrative provided by the family carers to generate the insights needed to address this research question. This has important implications for the design and execution of future research in this area. 

The burden on carers emerged as an area that needed further consideration, with an evident risk of overloading family members and carers in some cases. The amount of available carer time and commitment was an issue raised by three of the carers who emphasized the demands of overseeing the care and maintenance of the dog. It was noted by some that the time and energy involved in participation in the program had not been part of the original briefing, and this was further exacerbated for those carers who were also working outside the home. The issue of keeping the house free of dog hair was raised frequently, and three carers also reported that taking the dog for long trips in the car was “*more trouble than it was worth*”. Michelle (CS#3) observed that leaving the house with the dog was “*like travelling with a baby because of all the equipment*”. Future programs need to ensure families are well briefed, especially where they might not have had previous experience of owning a dog.

Veterinary and day-to-day costs were highlighted as an issue of wellbeing by several carers. At the time of this program, the Australian National Disability Insurance Scheme (NDIS) was not yet providing funding for assistance dogs for dementia participants. Being seriously out-of-pocket was of considerable concern, especially if providing care meant breadwinners were otherwise unable to work. Prescription foods, regular veterinary checks, vaccinations and so on proved costly, and medication and veterinary costs if the dog became ill sometimes proved to be prohibitive. In some cases, however, local veterinary practices chose not to charge at all for the care of assistance dogs. 

Lynette (CS#7) raised a concern about the accessibility of follow-up training when it was needed, or when they had questions about working with the dog. The paucity of available follow up appeared to impact her wellbeing. She described it as feeling “*a little bit left*”, and suggested more regular contact with the dog trainer, as well as more frequent follow-up training, “*especially in the first six months*”. She observed:

“*So, I know … having the dogs for dementia is very new, but I think there are other programs with dementia dogs running and I’d be interested to know … I knew there was no research. I just mean it would be interesting to see how similar or different the approach was from handing the dog over*”.

The transition of participants with dementia into either respite or full-time care (CS#1, #2, & #3) required close liaison between the carers and the supporting organization and care facilities, and this proved to be an emotional burden for some carers. This is a new area of policy for many care facilities, and while some were well set up to have assistance dogs attend as part of respite care, others were very reluctant. Those that enabled the assistance dog to attend demanded a very high level of support from the carer as well as from the dementia support agency, but even with this support, it was found to be very problematic having the dog resident in the care facility. During this program, no dogs went into full-time care with their participant, but two attended on a regular basis at least several times a week, with the full-time attendance of the carer. 

Carer depression was noted as an issue that emerged regularly, but that was not necessarily evident consistently. The qualitative interviews suggested that the level of carer depression was tied much more to incidents related to the progression of dementia in their family members, than to the presence of the dog. Similarly, interviews suggested that sadness and grief were not necessarily consistently evident. This is something that warrants further exploration in carers of people with YOD. As Lynette (CS#7) noted:

*Obviously, the mental health of both of us is important, but what’s behind that is a huge amount of grief, and loss and guilt. They all get thrown in together. The grief is huge … there’s all the plans that you had. There’s both us having to leave jobs and friends behind … He was teaching music. Instrumental teaching … He misses playing and performing very much. So, you can talk about it in terms of depression and that kind of thing, and obviously depression can arise from all that, and has often done, but to me it’s more layered and more complex.*

For most of the carers, this sense of loss was nuanced, but at times unmanageable, even with the support of the assistance dog. However, as Chris (CS#8) commented: “*having the assistance dog is the best thing that has happened to us*”.

## 4. General Implications and Future Directions

In this study, we investigated the impact of assistance dogs on family carers of people with YOD. Though the sample was relatively small, the data were collected over a two-year period and were rich and nuanced. These data reveal an important story of a diverse range of experiences; the good and the challenging. Many of the participants’ experiences reflected and affirmed the findings of Ritchie et al. [13]. This was particularly notable in the development of the human–animal bond, the relationship dynamics among the triads, the flexible nature of responsibility for caring, and the overall impact on carer wellbeing. 

Concerns were raised, however, by carers about the time and energy required of carers, and the financial impact of having an assistance dog (e.g., veterinarian expenses, grooming and food) in the absence of the support of a publicly funded scheme such as the Australian NDIS. When considering the placement of an assistance dog, it is not only the wellbeing of the primary owner that needs to be taken into consideration and that of the anyone who is likely to take on a primary care role, and as a consequence the care of the dog, but also importantly the wellbeing of the dog and the family’s capacity to meet these needs [25]. Furthermore, several cases in this study demonstrated the importance of sustaining the triadic relationship between the person with dementia, their carer *and* the dog when the person with dementia moved to out-of-home care. Regrettably, none of the dogs were able to transfer full-time to the out-of-home care facilities. In an effort to keep owners and their dogs together (or at least in regular contact) for as long as possible, this is a policy and practice issue that needs to be addressed by out-of-home / aged care facilities.

A caveat of this research is the relatively small size of the sample. It also operated within a limited timeframe (albeit over two years). It would have been useful to be able to follow up the impact of the project on carers in the longer term. Additionally, it would have been useful to have further investigated how the assistance dogs managed both the demands of their working schedules on a long-term basis [27] and the subsequent separation from their primary carer. Future research could investigate both the longer-term impact on carers and the longer-term wellbeing of the dogs (including their care and access to veterinary services, where dogs needed to be retired from the program or where they stayed with the family in the absence of their primary partner when the person with YOD needed to go into residential care or died).

Since this project was completed, several participants with YOD have died, and others are now in long-term care. It would be valuable to have been able to formally follow up the impact of the assistance dogs on carers and families as circumstances changed over time. Anecdotally, Hugh died soon after the end of the study, and his assistance dog went to live with his son and grandchildren, as Hugh’s partner/carer Marlene felt sad having the dog without Hugh. The dog had enabled the rebuilding of interactions between Hugh and his grandchildren, so the placement of the dog with them may well have enabled further family support. Similarly, Jason died (of an unrelated illness), but Annabelle chose to keep the dog with her as it provided her with great support and comfort. Despite its limitations, at the time of completion, this research project constituted the largest and longest project of its kind, and certainly the only one focusing on the circumstances surrounding assistance dogs for both people with YOD and their family carers. 

## 5. Conclusions

Trained assistance dogs can play an important role in promoting the health and wellbeing of people with YOD and that of their family carers, both in terms of hedonic wellbeing and eudemonic wellbeing. There is evidence of a strong human–animal bond not only between the assistance dog and the person with dementia, but also with the primary family carer and other family members. The relationship dynamics enabled and enhanced by the dog as part of the triad aided the carer in the practical execution of their role and in sustaining the wellbeing necessary for them to continue that role. Notably, our data suggests the positive effects of the relationship between the carer (and the wider family) and the assistance dog can continue beyond the move of the person with YOD into out-of-home care. However, support needs to be in place as the circumstances of the family member with YOD changes and the role and place of the assistance dog as part of a triad also changes. Policies need to be developed to support the dogs to have greater access to nursing homes, so they might continue a regular relationship with the person with YOD and thus continue to deliver on the benefits of the human–animal bond. Practical support with ongoing care of the dog, including follow-up from experienced trainers, and financial support of a scheme such as the Australian NDIS could also be important. 

## Data Availability

Raw data not publicly available consistent with ethics approval. Inquiries to be directed to corresponding author, McVilly.
